# Homo-epitaxy and twinning produce complex nanostructures in cryogenic calcite

**DOI:** 10.1107/S1600576726001354

**Published:** 2026-03-26

**Authors:** Péter Németh, Marco Bruno, Dino Aquilano, Zsombor Molnár

**Affiliations:** a Institute for Geological and Geochemical Research HUN-REN Research Centre for Astronomy and Earth Sciences (MTA Centre of Excellence) Budaörsi út 45 Budapest 1112 Hungary; b Research Institute of Biomolecular and Chemical Engineering, Nanolab University of Pannonia Egyetem út 10 Veszprém 8200 Hungary; c Dipartimento di Scienze della Terra Università degli Studi di Torino Via Valperga Caluso 25 Torino 10125 Italy; d Centre for Nanostructured Interfaces and Surfaces Università degli Studi di Torino Via G Quarello 15/a Torino 10135 Italy; Instituto Andaluz de Ciencias de la Tierra, Granada, Spain

**Keywords:** homo-epitaxy, twinning, cryogenic calcite, complex nanostructures

## Abstract

Calcite is famous for the rich diversity of oriented crystal growths. Although these are commonly explained by twinning, there are cases where the relationship between the associated crystals is not related to axis or mirror plane symmetry. Here, using transmission electron microscopy (TEM), we investigate the nanostructures of cryogenic calcite samples from Oknothichya (Hunter’s) cave, Baikal area (Russia), and demonstrate the homo-epitaxial intergrowth of {1120}//{1120}_rotated_, {0001}//{1100} and {1102}//{1108} calcite interfaces. We document as well the occurrence of extra calcite reflections at positions halfway between the  and  Bragg reflections in electron diffraction patterns and the doubling of  and  spacings (corresponding to 2 × 2.50 Å and 2 × 3.85 Å, respectively) in high-resolution TEM images. These features are similar to those associated with *c*-type reflections, and here we demonstrate that they are unrelated to Ca–Mg ordering as we study pure calcite samples. We also present a complex high-resolution TEM image that provides evidence for the cooperation of {1014} calcite twins with homo-epitaxial intergrowth at {1120}//{1120}_rotated_ and {1102}//{1108} interfaces in a subglacially formed calcite from Elephant Moraine (Antarctica). We develop structure models for all the recognized interfaces and report that they are energetically favorable.

## Abstract 2

A transmission electron microscopy study of cryogenic calcite samples reveals the homo-epitaxial intergrowths of {1120}//{1120}_rotated_, {0001}//{1100} and {1102}//{1108} interfaces, all being energetically favorable, along with their cooperation with {1014} twinning.

## 1. Introduction

Distinguishing between twinning and epitaxy is essential for correctly interpreting the crystallographic relationships and the processes that form either minerals or synthetic materials. Although both involve oriented crystal growth, their origins and structural implications differ fundamentally. Twinning occurs when two or more individuals of the crystalline phase (*A*) can be brought to the same orientation by rotation or reflection, by means of symmetry elements that do not belong to the symmetry of the crystalline phase (*A*) (Friedel, 1926 ▸; Ferraris *et al.*, 2004 ▸). Twinning may arise during growth, deformation or transformation within a crystal (*A*). In contrast, epitaxy occurs when crystal (*A*) (the deposit) grows on the surface of crystal (*B*) (the substrate) (Aquilano *et al.*, 2023 ▸). Crystals (*A*) and (*B*) do not necessarily have the same composition and structure, and the lattice matching at the interface controls the process. A special case is homo-epitaxy, the oriented intergrowth of two (or more) different forms {*hkl*} and {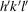} of the same crystal species (*A*). As for twinning, a crystallographic relationship occurs between the forms {*hkl*} and {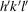}, but this relationship does not coincide with a symmetry element (axis or mirror plane). Confusing twinning with epitaxy can therefore lead to misinterpretation of formation conditions, interfacial energetics or even phase identification. Distinguishing between these two options provides key insights into the material’s genetic history and properties in both natural and engineered systems.

Calcite is the thermodynamically stable CaCO_3_ polymorph at ambient conditions and is famous for its wide variety of oriented intergrowths (Goldschmidt, 1913 ▸; Richards, 1999 ▸; Bruno *et al.*, 2010 ▸; Aquilano *et al.*, 2023 ▸; Aquilano *et al.*, 2024 ▸). It is rhombohedral (unit-cell parameters *a* = 4.989 and *c* = 17.06 Å, space group 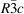) and the most frequent twin laws include {0001}, {0112}, {1014} and {0118} (Richards, 1999 ▸; Bruno *et al.*, 2010 ▸). The twin domain size ranges from several centimetres down to a few nanometres, and they may form by deformation (Barber & Wenk, 1979 ▸; Burkhard, 1993 ▸), growth (*e.g.* Larsson & Christy, 2008 ▸) and/or transformation (*e.g.* Németh, 2021 ▸). Homo-epitaxial relationships between {0112} and {0118} rhombohedra, as well as the {1014} rhombohedron and the basal {0001} pinacoid, have been studied at a theoretical level by Aquilano *et al.* (2024 ▸). In this study, as in others just mentioned, both twins and homo-epitaxies have been investigated in pure geological samples; this is to avoid any confusion with other calcite twins, as obtained elsewhere by the mediation of organic molecules (Pokroy *et al.*, 2007 ▸).

Transmission electron microscopy (TEM) is well suited for identifying intergrown calcite domains. In particular, high-resolution TEM (HRTEM) images can reveal lattice fringes from individual domains, and selected-area electron diffraction (SAED) patterns can display reflections arising from superimposed reciprocal lattices of the domains. The superposition is related to the presence or absence of symmetry operators, depending on whether twinning or homo-epitaxy occurs. Németh (2021 ▸) reported that {1014} twinning and the orientation change of the carbonate groups across the twin interface can double the *d_hkl_* spacings and result in extra reflections relative to a single crystal, which can be confused with ordering and erroneously attributed to superstructures. In particular, the occurrences of the *c*-type reflections (*l* = 2*n* + 1 for 0*kl* reflections) were attributed to various superstructures in Mg-bearing calcite and dolomite (Reeder & Wenk, 1979 ▸; Van Tendeloo *et al.*, 1985 ▸; Wenk *et al.*, 1991 ▸). Similar features, however, may also arise from homo-epitaxy. Indeed, in a SAED study of sea urchins, Larsson & Christy (2008 ▸) documented several calcite intergrowths yielding *d_hkl_* spacings and reflections inconsistent with those of 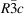 calcite. Although these features were explained by calcite twin individuals hosted within the calcite matrix, their characteristics are consistent with the homo-epitaxial relationship of calcite domains.

Here, using TEM, we examine cryogenic samples formed in Oknothichya (Hunter’s) cave, Baikal area (Russia) and demonstrate the homo-epitaxial intergrowth at {1120}//{1120}_rotated_, {0001}//{1100} and {1102}//{1108} interfaces, resulting in 5.00 and 7.70 Å distances and corresponding to doubled 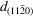 and 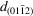 spacings. The samples were chosen to provide insights into the replacement structure of a water-rich cryogenic mineral, ikaite (calcium carbonate hexahydrate), that transforms to calcite above 5 °C. Although no diagnostic ikaite structural relicts were detected, similar to what was reported previously (Németh *et al.*, 2022 ▸), the homo-epitaxial intergrowth found can be generalized to calcite, grown in any samples/environments. In fact, the identified characteristic features are similar to those associated with *c*-type reflections, and here we demonstrate that they are unrelated to Ca–Mg ordering, as we study practically pure calcite samples. We document, as well, a complex HRTEM image that provides evidence for the cooperation of {1014} calcite twins with homo-epitaxial intergrowth at {1120}//{1120}_rotated_ and {1102}//{1108} interfaces in a subglacially formed calcite from Elephant Moraine (Antarctica) (Frisia *et al.*, 2025 ▸). Using geometry optimization, we develop structure models and demonstrate that these (complex) intergrowth types are energetically favorable.

## 2. Experimental

### 2.1. Samples and TEM investigation

A powder sample from Oknothichya (Hunter’s) cave in the Baikal region was provided by Yuri Dublyansky (University of Innsbruck, Austria) and Olga Kadebskaya (Mining Institute, Perm, Russia). The sample was originally collected in the ice cave as ikaite that transformed into calcite upon removal from the cave and exposure to surface temperatures (Bazarova *et al.*, 2014 ▸). The powder was crushed in ethanol, and its suspension was deposited onto copper grids covered by Lacey carbon supporting films. The process of sample crushing under ethanol in an agate mortar was short (1 min), during which time we did not expect to induce structural changes in the sample. According to literature data (*e.g.* Jamieson & Goldsmidt, 1960 ▸; Criado & Trillo, 1975 ▸), structural changes in carbonates occur as a result of long (several hours) aggressive mechanical milling/grinding, which are not comparable to our TEM sample preparation. Furthermore, the characteristic features of our TEM data match with previous studies prepared via ion-beam thinning (Van Tendeloo *et al.*, 1985 ▸). Therefore, we consider the nanostructures that we report to be pristine. Bright-field TEM (BFTEM), HRTEM and SAED data were acquired with a 200 kV Talos Thermo Scientific electron microscope. Energy-dispersive spectrometry (EDS) was performed with a ‘Super-X’ detector system built into the Talos F200X microscope column.

A BFTEM image of a focused ion beam prepared lamella from a black calcite sample, PR13081, collected at the Elephant Moraine site in Antarctica, was reported as Fig. 3(*c*) by Frisia *et al.* (2025 ▸). The image showed a black line parallel with the {1014} calcite plane. From the central part of the black line area, HRTEM images were obtained with a Thermo Fisher Scientific FEI THEMIS 200 (aberration-corrected) microscope operating at 200 kV accelerating voltage. Below, we analyze the HRTEM image obtained from the area adjacent to that shown in Fig. 6(*a*) of Frisia *et al.* (2025 ▸) and interpreted as {1014} twins.

**Figure 1:**
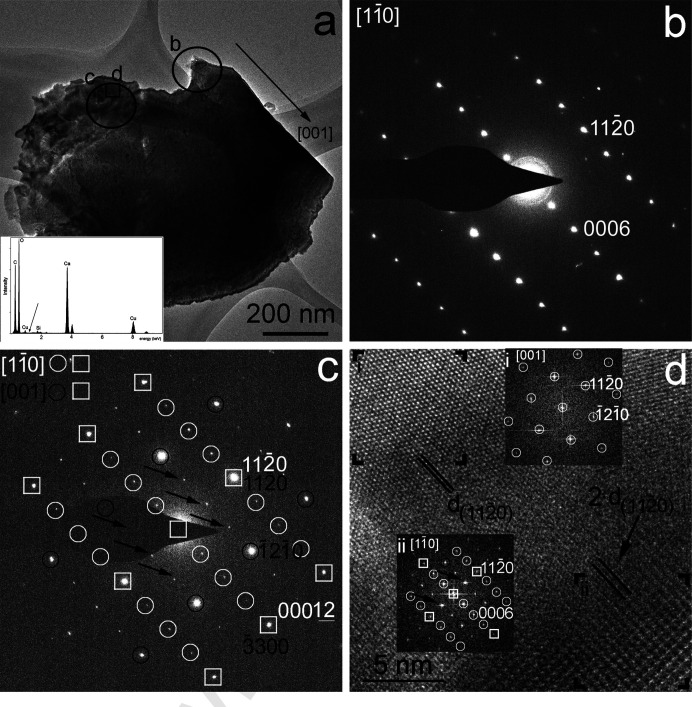
Intergrowth of calcite domains, projected along 〈110〉 and 〈001〉 and suggesting homo-epitaxy between {1120}//{1120} and {0001}//{1100} interfaces. (*a*) BFTEM image of a grain from the Okhotnichya cave calcite displaying sharp and rugged edges. A black arrow marks the *c* crystallographic direction of calcite. EDS data, shown in the lower left corner, confirm the sample is pure calcium carbonate. The Mg *K*α line (black arrow) is under the detection limit (0.5 m%). (*b*) SAED pattern taken from the circled area marked ‘b’ in (*a*) and its interpretation as calcite viewed along 〈110〉. (*c*) SAED pattern taken from the circled area marked ‘c’ in (*a*) and its interpretation as calcite domains viewed along 〈110〉 and 〈001〉. Overlapping reflections are marked by white rectangles, and reflections belonging to one domain only are marked by open black and white circles. Black arrows point to reflections with 5.0 Å spacing corresponding to doubled 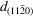 spacing. (*d*) Selected regions of the HRTEM image, taken from the rectangle area marked ‘d’ in (*a*), display 2.50 and 5.00 Å spacing corresponding to 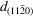 and doubled 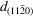 spacing, respectively.

Fast Fourier transforms (FFTs) obtained from the HRTEM images were calculated using Gatan *DigitalMicrograph* 3.6.1 software. The semi-quantitative EDS analysis of the grains from Oknothichya cave [Figs. 1 ▸(*a*) and 2 ▸(*a*)] and the HRTEM images of the black calcite sample (Frisia *et al.*, 2025 ▸) showed that they contain only Ca, O and C atoms.

### 2.2. Structure optimization and modeling

The homo-epitaxy in calcite was investigated at an empirical level. On the basis of the TEM results a composite calcite slab, (*hkil*)//(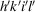), was generated (Bruno *et al.*, 2015 ▸; Bruno *et al.*, 2017 ▸) in the following way: (i) we searched for the two-dimensional coincidence lattices (2D-CLs hereinafter) between (*hkil*) and (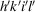) faces of the calcite (Table 1 ▸), in epi-relationship at a reticular level; (ii) (*hkil*) and (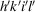) slabs of a selected thickness were constructed by cutting the bulk structure of calcite parallel to the lattice planes of interest and using the same 2D-CL parameters describing the found epitaxy; (iii) the (*hkil*) slab was placed above the (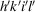) slab; (iv) finally, the composed slab structures (atomic coordinates and 2D-CL parameters) were optimized by considering all the atoms as free to move.

A 90° horizontal rotation was considered between the {1120} slabs, and one of the slabs was referred to as {1120}_rotated_. Structure optimization of the (1120)//(1120)_rotated_ and (0001)//(1100) composed calcite slabs has been performed at an empirical level by using the Ca-carbonate force field (Rohl *et al.*, 2003 ▸) along with version 4.0 of the *GULP* simulation code (Gale, 1997 ▸). The (0001) surface can be Ca-terminated or CO_3_-terminated, (0001)_Ca_ and (0001)_CO3_; then two different configurations of the (0001)//(1100) interfaces were simulated. The computational parameters we adopted are suitable to guarantee convergence on the energy values discussed in the main text, as well as the thickness of the composed slab. *GULP* output files, listing the optimized fractional coordinates along with the optimized 2D-CL parameters, are freely available at https://marco-bruno.weebly.com/download.html. We only performed static calculations at 0 K, the vibrational entropy and energy not being calculated. However, as previously discussed (Bruno *et al.*, 2013 ▸; Bruno, 2015 ▸), neglecting the vibrational contribution should not lead to a significant error in estimating the thermodynamic quantities (β and γ) described below. A detailed description of the computational methodology used for the interfaces has already been published (Bruno *et al.*, 2015 ▸; Bruno *et al.*, 2017 ▸).

The adhesion energy 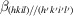 (erg cm^−2^) reads
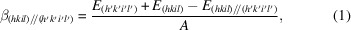
where 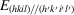, 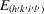 and 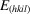 represent the energies of the composed (*hkil*)//(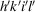) and isolated (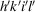), (*hkil*) slabs, respectively, and *A* is the area of the 2D-CL. Moreover, 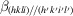 is related to the specific interface energy 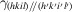 (erg cm^−2^) by Dupré’s relation (Kern, 1978 ▸):
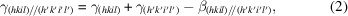
where 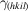 and 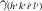 are calculated in the vacuum of the (*hkil*) and (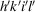) faces, respectively.

## 3. Results and discussion

### 3.1. Homo-epitaxially intergrown {1120}//{1120} interfaces

**Figure 2:**
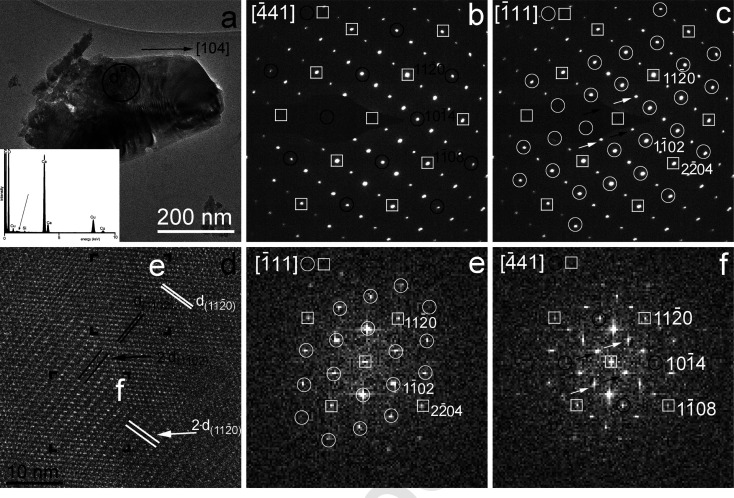
Intergrowth of calcite domains, projected along 〈441〉 and 〈111〉, suggesting homo-epitaxies between {1120}//{1120} and {1108}//{1102} interfaces. (*a*) BFTEM image from Okhotnichya cave calcite. The right upper corner of the grain shows sharp edges parallel to 〈104〉 direction of calcite. The central portion of the grain displays a mottled texture. EDS data, shown in the lower left corner, confirm the sample is pure calcium carbonate. The Mg *K*α line (black arrow) is under the detection limit (0.5 m%). (*b*) and (*c*) SAED patterns taken from the black circled area of (*a*) and its interpretation as calcite domains viewed along 〈441〉 and 〈111〉. White rectangles mark overlapping reflections, and reflections belonging to one domain only are marked by open black and white circles. Black and white arrows point to reflections with 7.70 and 5.00 Å spacing corresponding to doubled 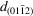 and 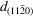 spacings, respectively. (*d*) Selected regions of the HRTEM image, taken from the rectangular area marked ‘d’ in (*a*), display 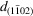, doubled 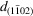, 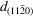 and doubled 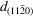 spacings. (*e*) and (*f*) are FFTs calculated from regions ‘e’ and ‘f’ of (*d*) and their interpretation as calcite viewed along 〈111〉 and 〈441〉.

**Figure 3:**
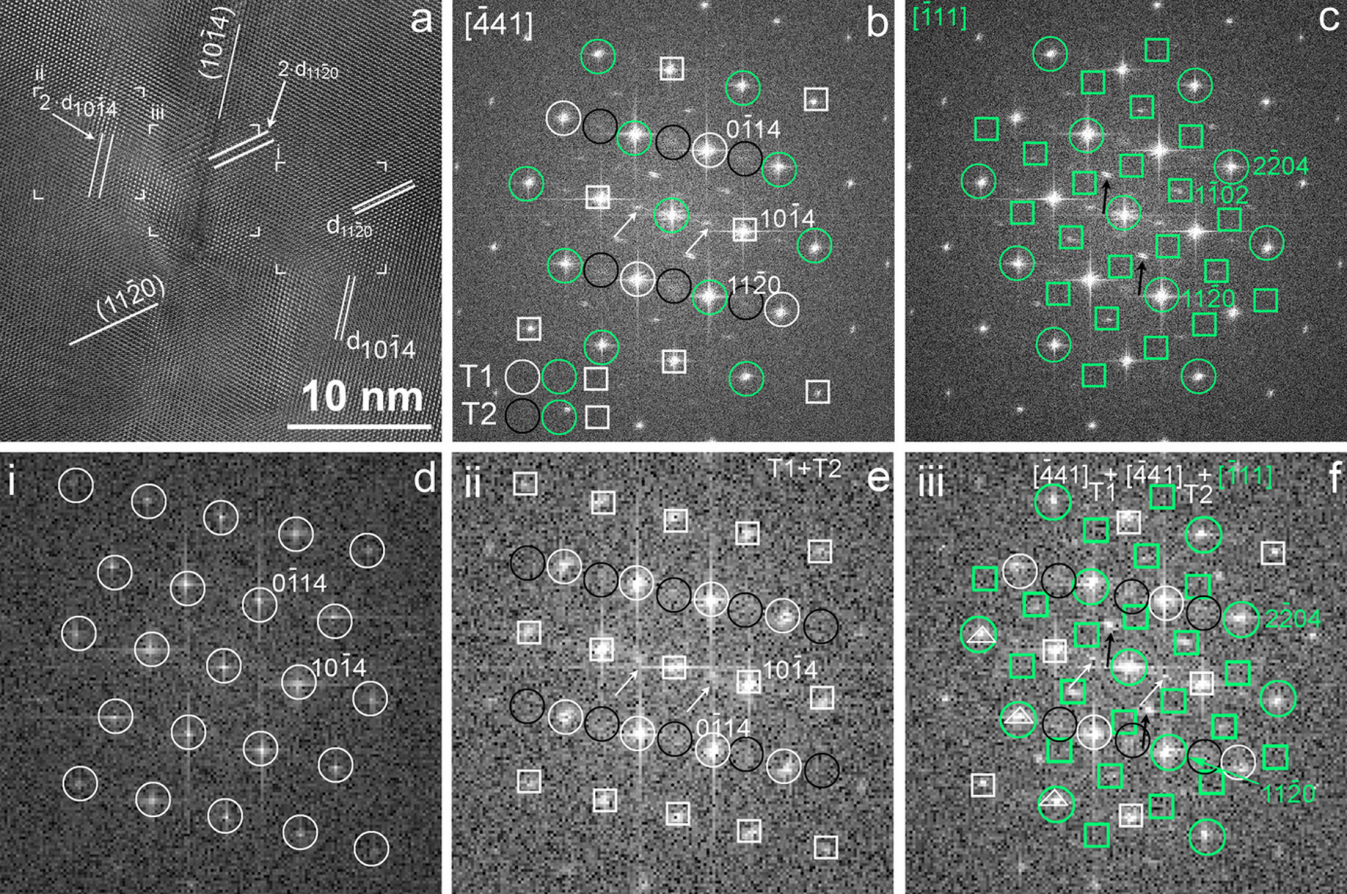
{1014} twinned calcite projected along 〈441〉 and its intergrowth with a domain projected along 〈111〉 suggests homo-epitaxy between {1120}//{1120} and {1108}//{1102} interfaces. (*a*) Complex HRTEM image from sample PRR13081, a subglacial calcite from the East Antarctic Ice Sheet. Fringes with 6.08 and 5.00 Å spacing corresponding, respectively, to doubled 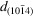 and 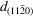 spacings are marked by white arrows. (*b*) FFT calculated from (*a*) and its interpretation as {1014} twinned calcite domains. White squares and open green circles mark overlapping reflections, and reflections belonging to one domain only are marked by open black and white circles. (*c*) FFT calculated from (*a*) and its interpretation as a domain projected along 〈111〉. Green open circles mark overlapping reflections between 〈441〉 and 〈111〉 domains. (*d*, *e* and *f*) FFTs calculated from regions i, ii, and iii of (*a*) and their interpretation as calcite, {1014} twinned calcite and {1014} twinned calcite intergrown with a domain projected along 〈111〉, respectively. Black and white open circles and green squares mark reflections arising from the individual domains. Overlapping reflections of the twinned domains and those arising from all domains are marked by white squares and green circles, respectively. White and black arrows mark reflections with doubled 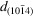 and 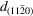 spacings.

The TEM study documents that the cryogenic samples have complex nanostructures (Figs. 1–3). In particular, BFTEM images and SAED patterns of the Oknothichya cave samples show heterogeneous contrast distributions [Figs. 1 ▸(*a*) and 2 ▸(*a*)] and reflections indicating an intergrown structure. Although the upper right corner of grain No. 1 is consistent with single-crystal calcite viewed along 〈110〉 [Fig. 1 ▸(*b*)], the SAED pattern obtained adjacent to the corner shows extra reflections halfway between the 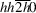 Bragg reflections [Fig. 1 ▸(*c*)]. We note that an SAED pattern with similar reflection distribution was reported by Larsson & Christy (2008 ▸). Interestingly, the intensities of the hexagonally arranged reflections with 2.50 Å spacing are systematically strong, indicating domain structure. In fact, this reflection distribution is consistent with calcite projected along the 〈001〉 direction. Considering the superposition of calcite domains projected along 〈110〉 and 〈001〉, a large portion of the reflections of Fig. 2 ▸(*c*) can be explained. The remaining reflections may be associated with electrons dynamically scattered from these superimposed domains or the composite interface of the intergrown domains. In either case, the intergrowth results in the superposition of the 1120 and 1120 as well as the 3300 and the 00012 reflections of the two domains, which we interpret as the homo-epitaxial intergrowth of {1120}//{1120} and {0001}//{1100} calcite interfaces.

To provide insights into the intergrown structure, we obtained an HRTEM image from the area marked ‘d’ on Fig. 1 ▸. Hexagonally arranged lattice fringes with 2.50 Å spacing occur on the upper left corner of this image, and its corresponding FFT shows reflections consistent with calcite projected along 〈001〉 [Fig. 1 ▸(*d*)]. In contrast, fringes with doubled 2.50 Å spacing, corresponding to doubled 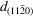 spacings, occur on the lower right area of the HRTEM image, and its corresponding FFT shows reflections consistent with intergrown calcite domains projected along 〈110〉 and 〈001〉 [Fig. 1 ▸(*d*)].

### 3.2. Homo-epitaxially intergrown {1102}//{1108} interfaces

Fig. 2 ▸ provides an example of an additional intergrowth type. Similar to Fig. 1 ▸(*a*), the BFTEM image of Fig. 2 ▸(*a*) shows undulating contrast. Although the upper right corner of the grain seems homogeneous, *i.e.* it may be a single crystal, this proposal cannot be confirmed since no SAED pattern was obtained from this region. The SAED patterns of Figs. 2 ▸(*b*) and 2 ▸(*c*) show extra reflections halfway between the 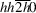 and 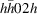 Bragg reflections, and we interpret these patterns as the intergrowth of calcite domains. This intergrowth results in the superposition of the 1120 and 1120 as well as the 1108 and the 2204 reflections of the two domains, which we interpret as the homo-epitaxial intergrowth of {1120}//{1120} and {1108}//{1102} calcite interfaces.

We studied the intergrowth with an HRTEM image obtained from the area marked ‘d’ in Fig. 2 ▸(*a*). Cross fringes with 2.50 and 3.86 Å spacings occur in the upper right corner, and the corresponding FFT shows reflections consistent with calcite projected along 〈111〉 [Fig. 2 ▸(*e*)]. In contrast, fringes with doubled 2.50 Å and 3.85 Å spacings, corresponding to doubled 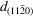 and 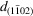 spacings, occur on the lower left area of the HRTEM image, and its corresponding FFT shows reflections consistent with intergrown calcite domains projected along 〈441〉 and 〈111〉 [Fig. 2 ▸(*f*)]. We note the doubled 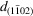 spacings were attributed to ‘*c*’ domains [Fig. 5(*b*) of Van Tendeloo *et al.* (1985 ▸)] and associated with Ca–Mg ordering in a dolomite sample, but here we demonstrate they can be explained by homo-epitaxy.

### 3.3. {1014} twinning with the cooperation of homo-epitaxial intergrowth at {1120}//{1120} and {1102}//{1108} interfaces

Fig. 3 ▸ documents the case of a complex intergrowth between twinning and homo-epitaxy from a subglacial-formed calcite, found in Elephant Moraine (Antarctica). The HRTEM image [Fig. 3 ▸(*a*)] is obtained from the area adjacent to that shown in Fig. 6(*a*) of Frisia *et al.* (2025 ▸), which was interpreted as {1014} twins. An inclined straight feature parallel to the calcite {1014} plane between the left and right sides of the image can indeed be recognized. However, the mirror plane related orientation change of the fringes with 2.50 Å spacing, corresponding to 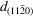 spacing of the 〈441〉 domains, is hidden. The twin is shown by the doubled 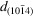 spacings and the occurrence of reflections halfway between the 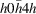 Bragg reflections in the FFT [Fig. 3 ▸(*b*)]. It was proposed that the vertical projection of small (<10 nm) twin domains hosted in an underlying calcite matrix and electrons dynamically scattered from these vertically stacked domains result in doubled 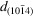 spacings (Németh, 2021 ▸). However, it is plausible that the unusual twin interface consisting of two {1014} layers with fundamentally different distortions (Yang *et al.*, 2024 ▸) explains the observed features.

{1014} twinning only partially explains the unusually complex HRTEM image [Fig. 3 ▸(*a*)] and its corresponding FFT [Fig. 3 ▸(*b*)]. Interestingly, a reflection occurs halfway between the 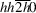 and 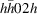 Bragg reflections. We showed above (Fig. 2 ▸) that these can be associated with the intergrowth of calcite domains projected along 〈441〉 and 〈111〉 [Fig. 3 ▸(*c*)] and the homo-epitaxies of {1120}//{1120} and {0001}//{1100} calcite interfaces. FFT calculations [Figs. 3 ▸(*d*)–3 ▸(*f*)] from various regions of the HRTEM image demonstrate the superposition of reflections arising from twinning and homo-epitaxy.

### 3.4. Structure models of the homo-epitaxial and twin interfaces

**Figure 4:**
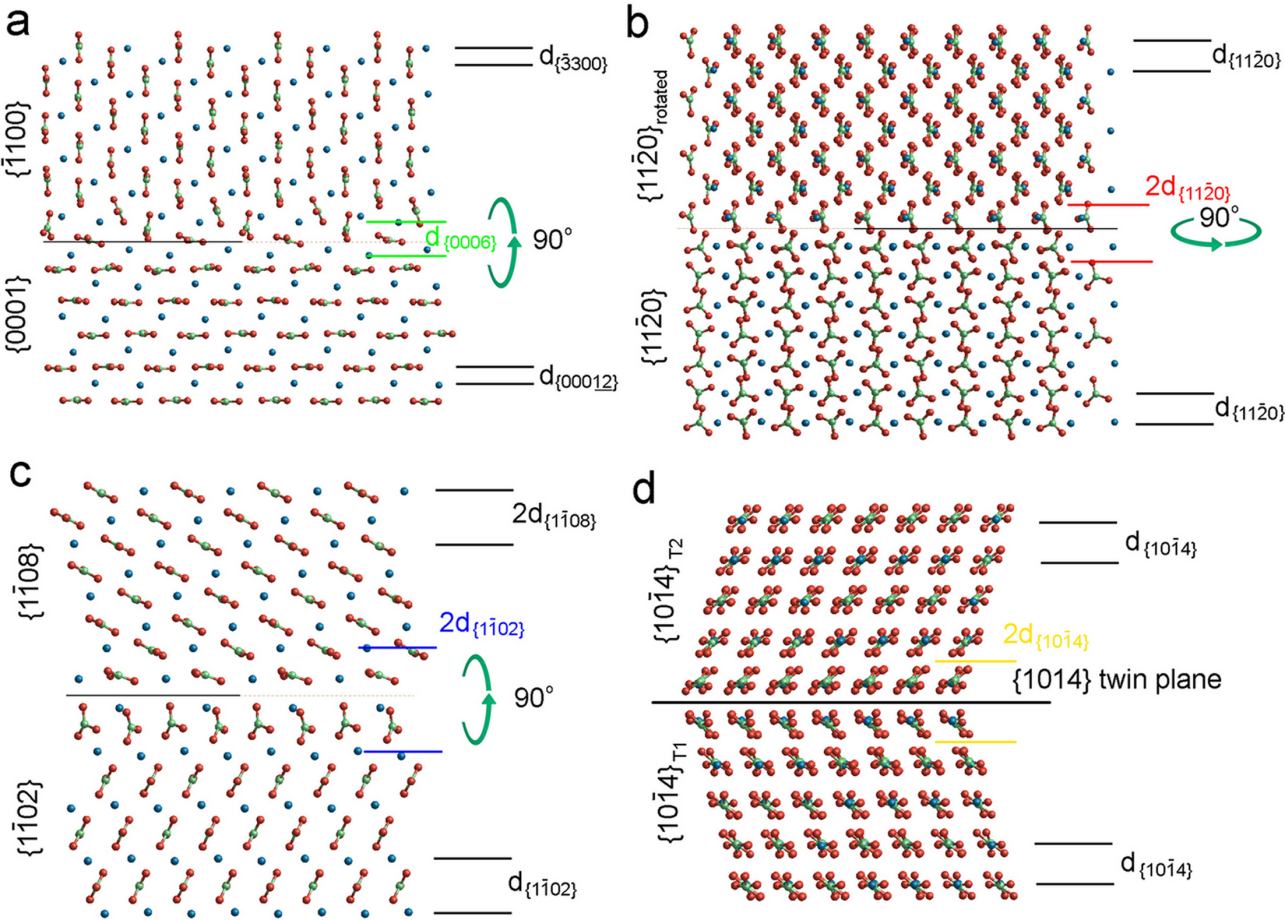
Geometry-optimized structure models of {0001}//{1100} (*a*), {1120}//{1120}_rotated_ (*b*), {1108}//{1102} (*c*) homo-epitaxial and {1014}//{1014} twinned (*d*) calcite interfaces. Blue, green and red balls represent Ca, C and O atoms, respectively. The relationships between the lower and upper slabs are as follows: vertical 90° rotation (*a*), horizontal 90° rotation (*b*), vertical 90° rotation (*c*), mirror plane (*d*). The 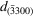 (1.440 Å) and the 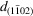 (3.855 Å) spacings closely match 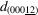 (1.422 Å) and 2 × 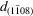 (3.824 Å), respectively. Doubled 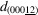 (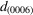), 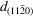, 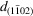 and 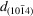 spacings are marked by green, red, blue and yellow lines, respectively.

The optimized structures of the interfaces 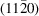//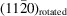, 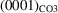//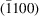, 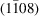//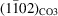 and 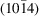//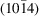 are drawn in Fig. 4 ▸. The interfaces of the (1102)//(1108) homo-epitaxy were previously studied by Aquilano *et al.* (2023 ▸), while the interface (1014)//(1014) describing the 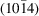 twin law was obtained by Bruno *et al.* (2010 ▸). The epitaxial interfaces of 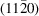//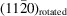 and (0001)//(1100) were first reported here on the basis of our TEM observations (Figs. 1 ▸–3 ▸ ▸). Their structure models were constructed by considering either vertical or horizontal rotations between the slabs [Figs. 4 ▸(*a*) and 4 ▸(*b*)], and the interface structures were optimized using the same calculation strategy and carbonate force field as described in previous work (Aquilano *et al.*, 2023 ▸; Bruno *et al.*, 2010 ▸).

The optimized 2D complex slabs (Fig. 4 ▸) of the various homo-epitaxial and (1014) twin interfaces reveal the rotation/shift of the CO_3_ groups and Ca atoms and demonstrate the coinciding *d* spacings of corresponding calcite planes between the different slabs. The intricate HRTEM images and SAED patterns previously described are not simply due to the superposition of the individual calcite slabs but also due to the structural modification of the composite interface. In fact, we hypothesize that the complex interface results in the doubled 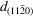 and 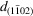 spacings (corresponding to 2 × 2.50 Å and 2 × 3.85 Å) (Fig. 4 ▸).

The β and γ values of the studied interfaces are listed in Table 2 ▸. The very low γ values associated with the (0001)_CO3_//(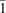
100), (1
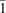
08)//(1
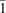
02)_CO3_ and (10
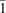
4)//(10
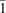
4) interfaces [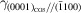 = 423 erg cm^−2^, 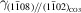 = 526 erg cm^−2^ and 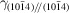 = 162 erg cm^−2^] suggest an elevated probability of observing these homo-epitaxial interfaces and 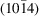 twins in calcite crystals. Although the high γ value [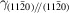
 = 1076 erg cm^−2^] suggests a lower probability of observing the 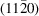//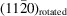 homo-epitaxy than that of the 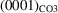//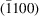 and 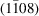//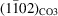 interfaces, our TEM data demonstrate their existence in natural calcite crystals.

### 3.5. The significance and the possible origin of the homo-epitaxial and twin intergrowth

We observed homo-epitaxial {1120}//{1120}_rotated_, {0001}//{1100} and {1102}//{1108} intergrowths in cooperation with {1014} twins in cryogenic samples. They may be associated with the cold ambient and/or the ikaite-to-calcite transition. It is plausible that the aqueous-solution supersaturation played a role during the transition (*e.g.* Molnár *et al.*, 2024 ▸), which may promote calcite intergrowth structures. However, the characteristic diffraction signatures of the intergrowth types are known from non-cryogenic formed calcite including biogenic samples (Larsson & Christy, 2008 ▸) as well as Mg-bearing calcite and dolomite (Reeder & Wenk, 1979 ▸; Van Tendeloo *et al.*, 1985 ▸; Wenk *et al.*, 1991 ▸) formed in temperate conditions; thus these intergrowths may widely occur in all kinds of geological samples. In fact, this proposal is supported by our calculation, which suggests the observed homo-epitaxial and twin interfaces are energetically favorable.

Our study calls for the reinvestigation of diffraction data that were previously attributed to *c*-type calcite reflections. We emphasize that such reflections are presumably unrelated to Ca–Mg ordering as they also occur in pure calcite samples and calcite homo-epitaxy provides a convincing explanation for their presence.

Here we demonstrate that even the {1120}//{1120}_rotated_ homo-epitaxial interface, which would be expected to have a low occurrence probability according to its relatively high calculated interface energy, is in fact present in natural samples. This strongly suggests that interface energies, while valuable as computed indicators, can be overridden in natural environments by other factors that modify their relative importance. Such new insights have the potential to substantially change how growth environments are inferred from calcite crystals in different geological settings.

## 4. Conclusion

TEM investigations of cryogenic calcite from a Baikal-area cave and a subglacial calcite from Antarctica showed lattice fringes with doubled 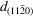 and 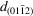 spacings (corresponding to 2 × 2.50 Å and 2 × 3.85 Å) and reflections at positions halfway between the 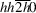 and 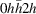 Bragg reflections. Although similar features have been associated with Ca–Mg ordering and various superstructures, these are not plausible explanations, as practically pure calcite samples were studied. The unusual features and the unique intensity distribution of the TEM data indicate domain structures with calcite projected along 〈110〉 and 〈001〉 as well as 〈441〉 and 〈111〉 directions. The crystallographic association of these domains results in the superposition reflections, which we interpret as various homo-epitaxial intergrowths of calcite interfaces. In particular we document the superposition of the 1120 and 1120 as well as the 3300 and the 00012 reflections, consistent with the homo-epitaxial intergrowth of {1120}//{1120} and {0001}//{1100} calcite interfaces. Our TEM data also indicate the homo-epitaxial intergrowth of {1120}//{1120}_rotated_ and {1108}//{1102} calcite interfaces, shown by the superposition of 120 and 1120 as well as the 1108 and the 2204 reflections. Furthermore, we demonstrate the cooperation of homo-epitaxial intergrowth at {1120}//{1120} and {1108}//{1102} interfaces with {1014} twinning, which result in an unusually complex HRTEM image.

To understand the structure of the observed homo-epitaxial and twin interfaces, 2D models were constructed and optimized by means of empirical calculations by considering the following crystallographic relationships:

(1) Vertical 90° rotation between the {0001} and {1100} slabs.

(2) Horizontal 90° rotation between the {1120} and {1120}_rotated_ slabs.

(3) Vertical 90° rotation between the {1108} and {1102} slabs.

(4) Mirror plane between the {1014} twinned slabs.

The geometry-optimized models showed rotation/shift of the CO_3_ groups and Ca atoms across the interfaces with negligible lateral mismatch between the slabs and demonstrated the coinciding *d* spacings of corresponding calcite planes between the different slabs. The complex interface structure was hypothesized to be responsible for the doubled 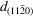 and 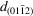 spacings. The low interface energies (γ) of the 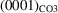//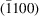, 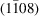//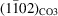 and 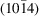//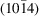 interfaces suggested an elevated probability of observing them in calcite crystals. Despite the higher γ value for 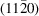//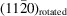 compared with the other interfaces, our TEM data confirmed its occurrence also.

Although we observed the various intergrowths in cryogenic samples, their occurrence can not necessarily be linked to cold environmental conditions: similar diffraction signatures have been observed in biogenic samples and Mg-bearing calcite and dolomite formed under temperate conditions. We presume these intergrowths may occur in a variety of geological samples. The hypothesis is further supported by calculations indicating that these homo-epitaxial and twin interfaces are energetically favorable.

## Figures and Tables

**Table:** 

	(0001)	(1100)	Linear and area misfit (%)
Vectors (Å)	[100] = 4.9814	[100] = 4.9814	0
	[430] = 17.2560	[001] = 17.0685	1.10
Area (Å^2^)	85.96	85.03	1.09

**Table:** 

	(1120)	(1120)_rotated_	Linear and area misfit (%)	Notes
Vectors (Å)	2 × [110] = 17.2560	[001] = 17.0685	1.10	90° horizontal rotation between slabs
	[001] = 17.0685	2 × [110] = 17.2560	−1.09	
Area (Å^2^)	294.53	294.53	0	

**Table:** 

	(1108)	(1102)	Linear and area misfit (%)	Notes
Vectors (Å)	[010] = 4.9814	[100] = 4.9814	0	Aquilano *et al.* (2023 ▸)
	1/3 × [841] = 12.8498	2/3 × [121] = 12.7494	0.79	
Area (Å^2^)	64.01	63.51	0.79	

**Table:** 

	(1014)	(1014)	Linear and area misfit (%)	Notes
Vectors (Å)	[010] = 4.9814	[010] = 4.9814	0	Bruno *et al.* (2010 ▸)
	1/3 × [421] = 24.2715	1/3 × [421] = 24.2715	0	
Area (Å^2^)	120.91	120.91	0	

**Table 2:** Adhesion (β) and interface (γ) energies expressed in erg cm^−2^ The (0001) and 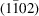 faces have two possible terminations: Ca-terminated, 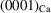 and 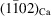, and CO_3_-terminated, 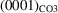 and 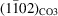. The interface energies were calculated by means of Dupré’s relation and the following surface energies: 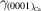 = 834, 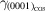 = 764, 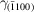 = 722, 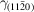 = 1232, 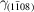 = 702, 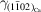 = 1040, 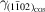 = 750 and 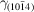 = 0.534 (Bruno *et al.*, 2008 ▸; Bruno *et al.*, 2010 ▸; Massaro *et al.*, 2010 ▸).

Interfaces	Adhesion energy (β)	Interface energy (γ)	Notes
Homo-epitaxy			
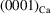 // 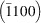	582	974	This work
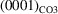 // 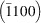	1063	423	This work
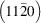 // 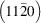	1388	1076	This work
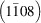 // 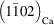	723	1019	Aquilano *et al.* (2023 ▸)
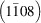 // 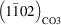	926	526	Aquilano *et al.* (2023 ▸)

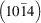 twin law			
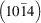 // 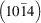	906	162	Bruno *et al.* (2010 ▸)

## Data Availability

*GULP* output files, listing the optimized fractional coordinates along with the optimized 2D-CL parameters, are freely available at https://marco-bruno.weebly.com/download.html.
